# IRAK-M has effects in regulation of lung epithelial inflammation

**DOI:** 10.1186/s12931-023-02406-5

**Published:** 2023-04-07

**Authors:** Jia Li, Zhoude Zheng, Yi Liu, Hongbing Zhang, Youming Zhang, Jinming Gao

**Affiliations:** 1 Department of Pulmonary and Critical Care Medicine, Peking Union Medical College Hospital, Chinese Academy of Medical Sciences and Peking Union Medical College, #1 Shuaifuyuan, Dongcheng District, Beijing, 100730 China; 2 Department of Respiratory Medicine, Civil Aviation General Hospital, Beijing, 100123 China; 3 Department of Physiology, Institute of Basic Medical Sciences, Chinese Academy of Medical Sciences and Peking Union Medical College, Beijing, 100730 China; 4 Section of Genomic and Environmental Medicine National Heart and Lung Institute, Imperial College London, London, SW3 6LY UK

**Keywords:** Asthma, Airway epithelial cells, Innate immunity, IRAK-M, CXCL10

## Abstract

**Background:**

Epithelial barrier is important for asthma development by shaping immune responses. Airway expressing-IL-1 receptor-associated kinase (IRAK)-M of Toll-like receptor pathway was involved in immunoregulation of airway inflammation through influencing activities of macrophages and dendritic cells or T cell differentiation. Whether IRAK-M has effect on cellular immunity in airway epithelial cells upon stimulation remains unclear.

**Methods:**

We modeled cellular inflammation induced by IL-1β, TNF-α, IL-33, and house dust mite (HDM) in BEAS-2B and A549 cells. Cytokine production and pathway activation were used to reflect the effects of IRAK-M siRNA knockdown on epithelial immunity. Genotyping an asthma-susceptible IRAK-M SNP rs1624395 and measurement of serum CXCL10 levels were performed in asthma patients.

**Results:**

IRAK-M expression was significantly induced in BEAS-2B and A549 cells after inflammatory stimulation. IRAK-M knockdown increased the lung epithelial production of cytokines and chemokines, including IL-6, IL-8, CXCL10, and CXCL11, at both mRNA and protein levels. Upon stimulation, IRAK-M silencing led to overactivation of JNK and p38 MAPK in lung epithelial cells. While antagonizing JNK or p38 MAPK inhibited increased secretion of CXCL10 in IRAK-M silenced-lung epithelium. Asthma patients carrying G/G genotypes had significantly higher levels of serum CXCL10 than those carrying homozygote A/A.

**Conclusion:**

Our findings suggested that IRAK-M has effect on lung epithelial inflammation with an influence on epithelial secretion of CXCL10 partly mediated through JNK and p38 MAPK pathways. IRAK-M modulation might indicate a new insight into asthma pathogenesis from disease origin.

**Supplementary Information:**

The online version contains supplementary material available at 10.1186/s12931-023-02406-5.

## Background

Our understanding of asthma immunopathogenesis has been advanced over the last decade [1]. Much evidence has pointed to the importance of airway epithelium in asthma inception [2]. Airway epithelium, as an immunological barrier, is thought to initiate and orchestrate airway inflammatory responses to environmental triggers through multiple mechanisms, one of which is airway-expressing Toll-like receptors (TLRs) pathway [3]. TLRs are important for early recognition of the inhaled environmental antigens, further triggering innate and adaptive immunity [3]. This process is tightly regulated by intracellular members of the IL-1 receptor-associated kinase (IRAK) family. IRAK-M, an inactive IRAK isoform, functions as a negative regulator of NF-κB signaling pathway dependent on the adaptor protein Myeloid Differentiation factor (MyD) 88 coupled with TLRs [4]. Interestingly, a recent study demonstrated that IRAK-M activation in Th2 airway inflammation induced by IL-33 was dependent on the prolyl cis–trans isomerase PIN1 mechanism [5].

Genetic studies have linked IRAK-M SNP variants to both early- and late-onset asthma, suggesting that airway-expressing IRAK-M may be involved in inappropriate activation of innate immunity in the bronchial cells [6, 7]. Aberrant expression of IRAK-M in airway epithelium has displayed the opposing role, anti- or pro-inflammatory effect, in mediating lung injury in the different context of the pathophysiological settings [8–11]. For example, IRAK-M expression was shown to be significantly elevated in the airway epithelial cells and mucosa of the experimental murine asthma models and asthmatic patients [7, 10, 12]. Our previous investigations reported that IRAK-M knockout exacerbated allergic airway inflammation via activation of macrophages and dendritic cells and promotion of Th2 immunity in the mouse models induced by OVA or house dust mite (HDM) [12, 13]. On the other hand, overexpression of IRAK-M showed proinflammatory effects. For instance, Wu et al. reported that persistent expression of IRAK-M induced by a Th2 cytokine IL-13 in asthmatic airway epithelial cells inhibited epithelial TLR2 defense mechanism [10]. Consistently, we used an IRAK-M knockout mouse model chronically exposed to OVA stimulation and showed that IRAK-M maintained Th2 airway inflammation and inhibited the DC-mediated Th1 activation [7]. Taken together, current evident supported IRAK-M involvement in the pathophysiology of lung inflammation mainly through activation of macrophages and dendritic cells or impact on T cell differentiation.

Given the multiple lines of evidence indicating that IRAK-M is involved in modulation of innate immunity, whether IRAK-M regulates airway epithelial inflammation and downstream signaling pathways has not been well understood. Here we performed in vitro investigations to test this possibility by examining the effect of IRAK-M knockdown on cellular immunity in immortalized lung epithelial cell lines under inflammatory stimulations. We found that IRAK-M showed effects on inflammatory events of lung epithelial cells, with an impact on Th1 cytokine CXCL10 production possibly through JNK and p38 MAPK pathways.

## Methods

### Cell culture

Human airway epithelial cell line BEAS-2B cells and human alveolar epithelial cell line A549 cells were purchased from Shanghai Institute of Life Sciences, Chinese Academy of Sciences (Shanghai, China). BEAS-2B cells were maintained in Dulbecco’s modified Eagle’s medium (DMEM) media (HyClone, USA) and A549 cells were cultured in F-12K media (Gibco, USA). All media were supplemented with 10% fetal bovine serum (Gibco) and 1% penicillin/streptomycin (HyClone). All cells were cultured at 37 °C in a humidified incubator of 5% CO_2_.

### siRNA-mediated IRAK-M knockdown

IRAK-M siRNA (si-IRAK-M; sense 5′-CCU-AAC-AUA-UGC-UGU-CAA-ATT-3′; antisense 5′-UUU-GAC-AGC-AUA-UGU-UAG-GTT-3′) were obtained from GenePharma (Suzhou, Jiangsu, China). Nontargeting pool-negative siRNA (si-con; sense 5′-UUC-UCC-GAA-CGU-GUC-ACG-UTT-3′; antisense 5′-ACG-UGA-CAC-GUU-CGG-AGA-ATT-3′) were used as controls. siRNAs transfections of BEAS-2B cells and A549 cells were performed using GP-transfect-Mate transfection reagents. siRNAs and transfection reagents were mixed following manufacturer’s protocol and incubated with the cells for 48 h. Cells were harvested with TRIzol reagent (Thermo Fisher Scientific, USA) for quantitative real-time polymerase chain reaction (qRT-PCR) or harvested with protein lysis buffer (5 ml 20% SDS, 0.77 g DTT (1 M), 3 ml Tris–HCl (1 M, pH 6.8), 5 ml 100% glycerol, 32 ml H2O) freshly added 1 mM phosphatase and protease inhibitor (Roche, Switzerland) for western blot.

### Airway epithelial cell model of inflammation

IL-1β (1 ng/ml; R&D system, USA), or TNF-α (10 ng/ml; R&D system), or IL-33 (100 ng/ml; R&D system), or HDM (10 μg/ml; Cosmo Bio USA, USA) was separately added to BEAS-2B or A549 cells. Cells were harvested at 0, 6, 12, 24 h for Western blot. BEAS-2B and A549 cells were starved in serum-free media for 24 h after IRAK-M siRNA transfection. The cell-free supernatant and cells were collected at 24 h after stimulation with IL-1β, or TNF-α, or IL-33, or HDM for analysis of cytokines. To reflect the effect of IRAK-M on the JNK and p38 MAPK signaling pathways in airway epithelial cells, BEAS-2B and A549 cells were harvested at 0, 10, 30, 60 min for Western blot after IRAK-M siRNA transfection. Cells were starved and stimulated. BEAS-2B and A549 cells were incubated with JNK inhibitor SP600125 (20 μM; MedChemExpress, USA) or p38 MAPK inhibitor SB203580 (10 μM; MedChemExpress) for 2 h. After 24 h of stimulation, cell-free supernatant and cells were harvested for cytokines analysis.

### Western blot

Whole-cell protein extracts were separated on 10% SDS–polyacrylamide gel electrophoresis gels and transferred to nitrocellulose filter membranes. The membrane was blocked with 5% non-fat dry milk in Tris-buffered saline (TBS) containing 0.1% Tween 20 (TBS-T) for 1 h. Then the membrane was cut and incubated with the respective primary Abs at 4 °C overnight. The Abs were as follows: anti-mouse IRAK-M, anti-mouse JNK, anti-mouse Phospho-JNK, anti-mouse p38 MAPK, anti-mouse Phospho-p38 MAPK and anti-mouse GAPDH (Cell Signaling Technology, USA). Primary Ab application was followed by incubation with HRP-conjugated secondary Abs (rabbit anti-mouse IgG) (Cell Signaling Technology) at room temperature for 1 h. After washing for three times with TBS-T, membranes were scanned using Odyssey CLx imaging system (LICOR, USA). Semi-quantitation was analyzed with Image J software (National Institutes of Health). Protein expression levels were normalized to GAPDH.

### ELISA

Commercial ELISA kits were used to measure the concentrations of the cytokines, including IL-6 (R&D system), IL-8 (R&D system), CXCL10 (R&D system and Laizee biotech, Shanghai, China), CXCL11 (R&D system), and IFN-γ (R&D system), in supernatants collected from cells.

### Quantitative real-time PCR

qRT-PCR was used to reflect mRNA expression of IRAK-M and cytokine and chemokines, including IL-6, IL-8, CXCL10, CXCL11 and IFN-γ. Total RNA prepared from the BEAS-2B or A549cells using TRIzol reagent was reverse transcribed into cDNA using the ALL-IN-ONE RT MasterMix (Applied Biological Materials, Canada). qRT-PCR was performed on the CFX Connect Real-Time PCR Detection System (Bio-Rad Laboratories, USA) in a 20 μl of final reaction volume containing 1 μl of cDNA and qPCR MasterMix (Applied Biological Materials). The relative quantities of mRNAs were calculated with the comparative cycle threshold method and normalized using human GAPDH as an internal control. The primer sequences are shown in Additional file 1: Table S1, as supplementary data.

### Participants

A total of 137 individual patients with asthma was recruited from Asthma Clinic of Beijing Aviation General Hospital between September 2021 and March 2022. Asthma was diagnosed by the respiratory specialists. All patients were fulfilled the diagnostic criteria of the Global Strategy for Asthma Management and Prevention [14]. The study was approved by the Ethics Committee for Human Research of Beijing Aviation General Hospital (MHZYY 2014-05-01). All participants provided informed written consent at enrolment.

### Genotyping

Genomic DNA was extracted from peripheral blood leukocytes by using TIANamp Genomic DNA Kit (TIANGEN BIOTECH, Beijing, China) according to manufacture protocol. Two SNPs of IRAK-M (rs1624395, forward 5′-ATGGGATTGGGAGAGAAGCC-3′ and reverse 5′- TAAGCCAAAAGCCAGGTCCA-3′; rs1370128, forward 5′-ACCATGGGGTCTGCATCATTT-3′ and reverse 5′-CTGGTTGGTTCTCCTGCAAC-3′) were genotyped by SNaPshot. The PCR products were sequenced on an ABI 3730xl DNA Analyzer (Applied Biosystems) and analyzed using the GeneMapper 4 software. The genotyping efficiency for IRAK-M SNP was > 95% and minor allele frequency was > 5%.

### Measurement of serum CXCL10 in asthma patients

Fasting blood samples were harvested from patients with asthma in the morning and centrifuged at 12,000 rpm. Serum was stored at − 80 °C until measurement. Serum CXCL10 concentrations were measured using a commercial ELISA kit (R&D system) following the manufacturer’s instructions.

### Statistical analysis

Data were expressed as mean ± SEM. Multiple comparisons were performed by one-way ANOVA with Tukey post-hoc test. Comparisons between two groups were made by unpaired Student *t*-test (GraphPad Prism Version 9.0, GraphPad, San Diego, CA). *P*-value < 0.05 was considered significant.

## Results

### IRAK-M induction in lung epithelial cells by multiple stimuli

**Fig. 1:**
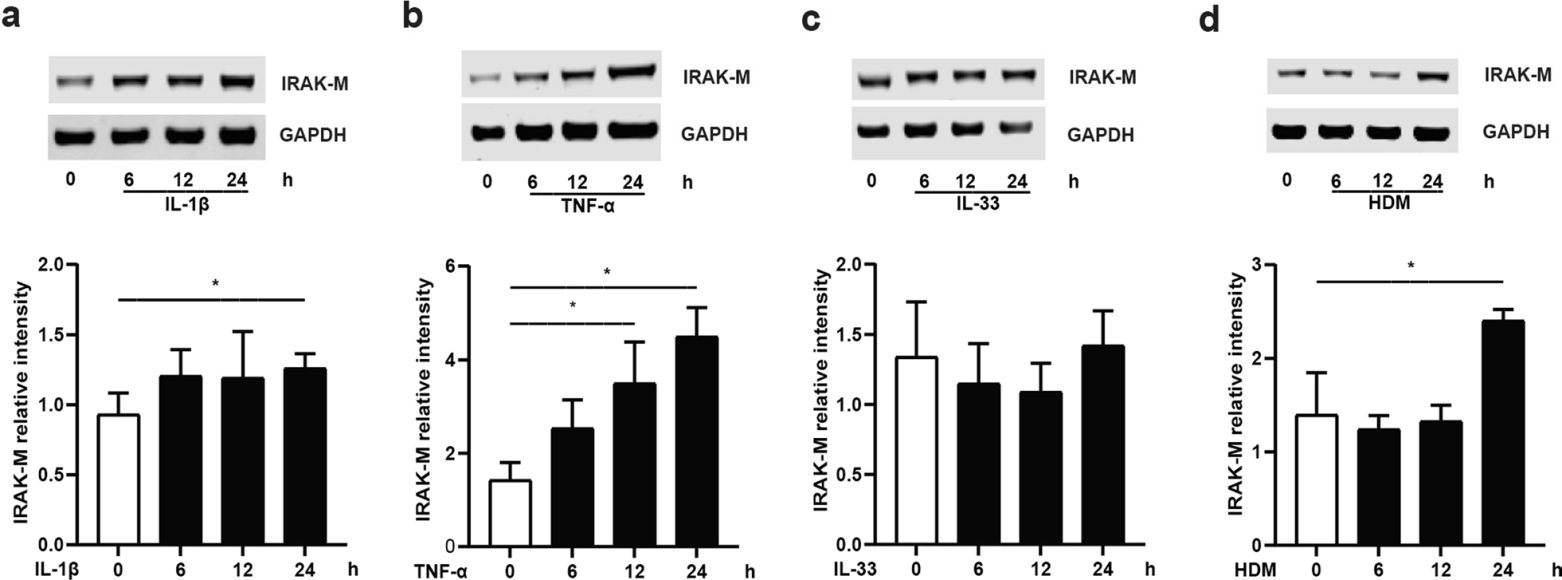
IRAK-M expression is inducible by multiple stimuli in BEAS-2B cells. IRAK-M protein expression at 0, 6, 12, 24 h after **a** IL-1β (1 ng/ml), **b** TNF-α (10 ng/ml), **c** IL-33 (100 ng/ml) and **d** HDM (10 μg/ml) exposure. IRAK-M protein expression was normalized to GAPDH. Values are expressed as mean ± SEM (n = 3). *P < 0.05

To investigate IRAK-M expression by lung epithelial cells in response to the exogenous stimuli, we performed a time course study to examine the dynamic change of IRAK-M protein in BEAS-2B and A549 cells after stimulation with IL-1β, or TNF-α, or IL-33, or HDM. IRAK-M protein was significantly induced by stimulation in BEAS-2B cells at indicated time points, except for IL-33 stimulation (Fig. 1a–d). The similar results were observed in A549 cells treated with the same manner (Additional file 2: Fig. S1a–d).

### Impact of IRAK-M knockdown on cytokines production in airway epithelial cells

To reflect the effect of IRAK-M on cellular inflammation in airway epithelial cells, IRAK-M knockdown was performed by RNA silencing. The expression of IRAK-M was significantly attenuated by IRAK-M siRNA at both mRNA and protein levels in BEAS-2B cells (Additional file 3: Fig. S2a) and A549 cells (Additional file 3: Fig. S2b).

**Fig. 2:**
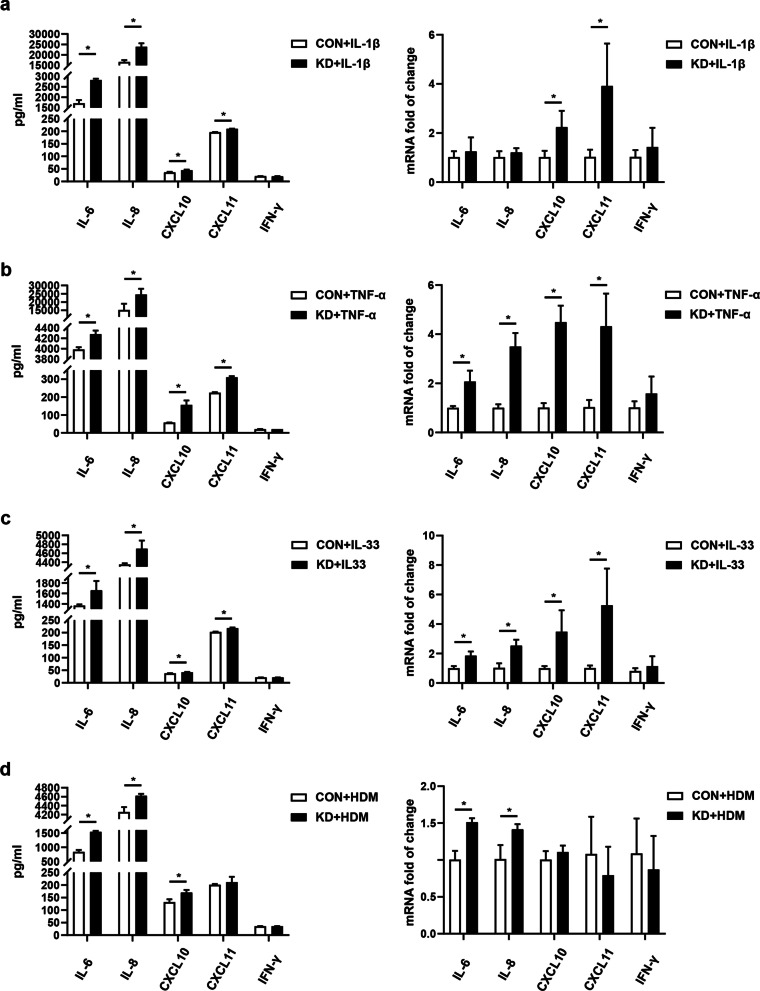
Impact of IRAK-M knockdown on cytokines production in BEAS-2B cells. Expression of IL-6, IL-8, CXCL10, CXCL11 and IFN-γ at both mRNA level and protein level after **a** IL-1β (1 ng/ml), **b** TNF-α (10 ng/ml), **c** IL-33 (100 ng/ml) and **d** HDM (10 μg/ml) stimulation for 24 h. mRNA expression of IL-6, IL-8, CXCL10, CXCL11 and IFN-γ were normalized to GAPDH. Values are expressed as mean ± SEM (n = 3). *P < 0.05. CON, si-control. KD, si-IRAK-M

IRAK-M knockdown significantly increased IL-1β-induced expression of IL-6, IL-8, CXCL10, and CXCL11 in BEAS-2B cells, whereas IRAK-M silencing had no significant impact on IL-1β-induced IFN-γ expression at both mRNA and protein levels (Fig. 2a). Similar results were observed in BEAS-2B cells stimulated by TNF-α, IL-33, and HDM (Fig. 2b–d), but IRAK-M knockdown had no effect on CXCL11 production after HDM stimulation (Fig. 2d).

To replicate the effect of IRAK-M on production of cytokines by alveolar epithelial cells, we examined cytokines expression at both mRNA and protein levels in A549 cells. IRAK-M knockdown significantly increased epithelial production of IL-6, IL-8, CXCL10, and CXCL11, rather than IFN-γ, in A549 cells treated with indicated stimuli (Additional file 4: Fig. S3a–d). IRAK-M knockdown had no effect on CXCL11 production after IL-33 stimulation (Additional file 4: Fig. S3c).

### Effect of IRAK-M on activation of JNK and p38 MAPK pathways in lung epithelial cells

**Fig. 3:**
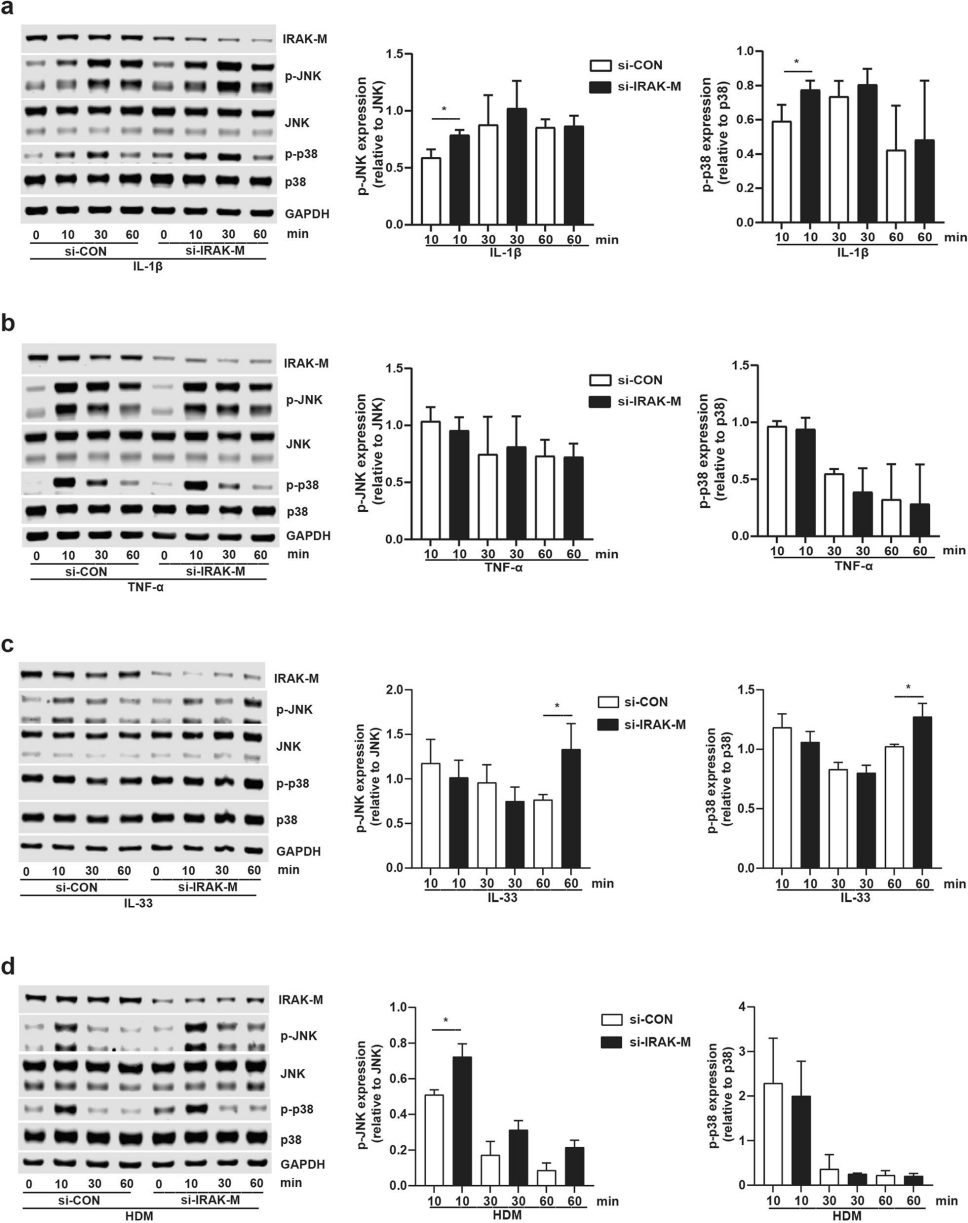
Effect of IRAK-M on activation of JNK and p38 MAPK pathways in BEAS-2B cells. Protein expression of p-JNK, JNK, p-p38, p38 after **a** IL-1β (1 ng/ml), **b** TNF-α (10 ng/ml), **c** IL-33 (100 ng/ml) and **d** HDM (10 μg/ml) stimulation for 10, 30 and 60 min. P-JNK was normalized to total JNK, while p-p38 was normalized to total p38. Values are expressed as mean ± SEM (n = 3). *P < 0.05

To reflect the effect of IRAK-M on the signaling pathways in airway epithelial cells, we examined the protein expression of phospho-JNK, JNK, phospho-p38 and p38 in BEAS-2B cells. IRAK-M knockdown caused elevation of phosphor-p38 and phosphor-JNK, protein expression, in cells after treatment with IL-1β, or IL-33 (Fig. 3a and c). IRAK-M silencing had no significant impact on phosphor-JNK and phosphor-p38 in BEAS-2B cells with TNF-α stimulation (Fig. 3b). IRAK-M knockdown caused increased protein expression of phospho-JNK, no p38 MAPK pathway activation, in cells after HDM stimulation (Fig. 3d).

We also examined the protein expression of phospho-JNK, JNK, phospho-p38 and p38 in A549 cells. Although IRAK-M silencing had no significant impact JNK and p38 MAPK pathway activation after IL-1β stimulation (Additional file 5: Fig. S4a), IRAK-M knockdown caused elevation of phospho-JNK and phospho-P38 protein expression in A549 cells stimulated with TNF-α, or IL-33, or HDM at indicated time points (Additional file 5: Fig. S4b–d).

### Influence of IRAK-M on CXCL10 expression and activation of JNK and p38 MAPK pathways in lung epithelial cells

**Fig. 4:**
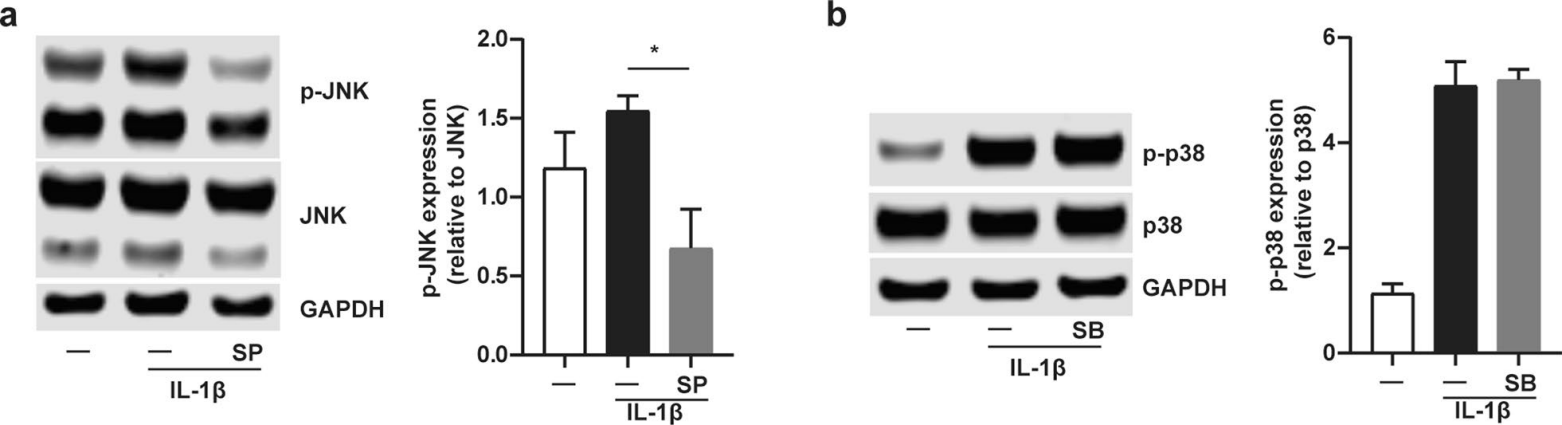
The activation of JNK and p38 MAPK pathway was blocked by inhibitor in BEAS-2B cells. **a** Protein expression of p-JNK and JNK after SP600125 (SP, 20 μM) pretreated for 2 h and IL-1β stimulated for 24 h. **b** Protein expression of p-p38 and p38 after SB203580 (SB, 10 μM) pretreated for 2 h and IL-1β stimulated for 24 h. P-JNK was normalized to total JNK, while p-p38 was normalized to total p38. Values are expressed as mean ± SEM (n = 3). *P < 0.05

We demonstrated that IRAK-M knockdown increased CXCL10 expression at both mRNA and protein levels in lung epithelial cells. Further, IRAK-M silencing caused activation of JNK and p38 MAPK pathway. To determine that whether IRAK-M had effect on CXCL10 expression by epithelial cells through JNK and p38 MAPK pathway, BEAS-2B cells were pretreated with SP600125 (SP, JNK inhibitor) and SB203580 (SB, p38 MAPK inhibitor) for 2 h and then exposed to IL-1β for 24 h. The JNK inhibitor significantly reduced the activation of JNK pathway (Fig. 4a), while the p38 MAPK inhibitor has no significant impact on the phospho-p38 protein expression in BEAS-2B cells (Fig. 4b). The similar results were observed in A549 cells pretreated with SP and SB (Additional file 6: Fig. S5a and 5b).

**Fig. 5:**
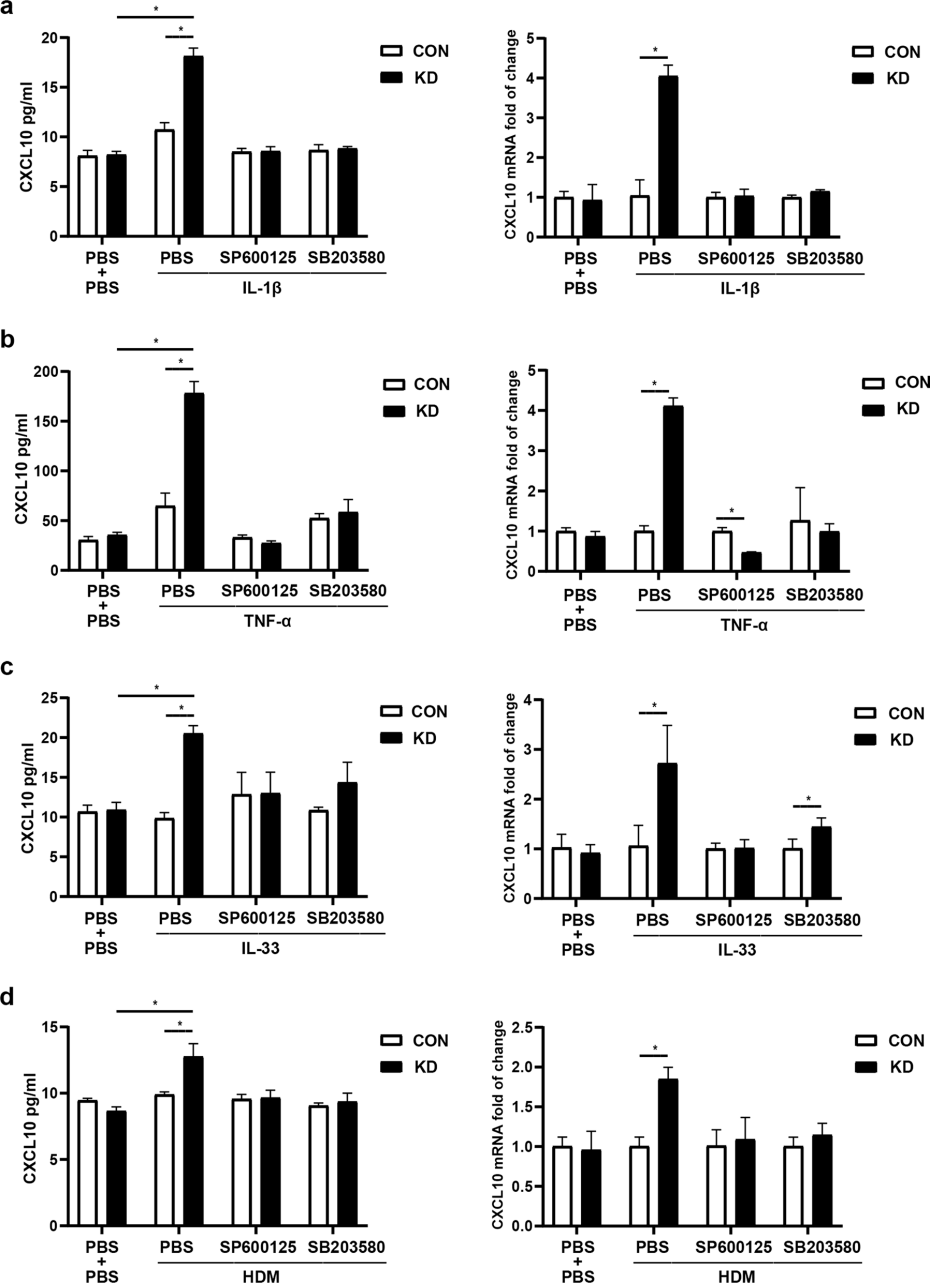
Treatment with JNK or p38 MAPK inhibitor attenuated IRAK-M knockdown-mediated CXCL10 secretion upon stimulation in BEAS-2B cells. Expression of CXCL10 at both mRNA and protein level after PBS, SP600125 (SP, 20 μM) and SB203580 (SB, 10 μM) incubation for 2 h and **a** IL-1β (1 ng/ml), **b** TNF-α (10 ng/ml), **c** IL-33 (100 ng/ml) and **d** HDM (10 μg/ml) stimulation for 24 h. Values are expressed as mean ± SEM (n = 3). *P < 0.05. CON, si-control. KD, si-IRAK-M

We also examined the effect of IRAK-M on CXCL10 expression in BEAS-2B cells incubated with JNK or p38 MAPK inhibitor. IRAK-M knockdown had no significant impact on the expression of CXCL10 at both mRNA and protein levels by cells after incubation with JNK or p38 MAPK inhibitor and stimulation with IL-1β or HDM (Fig. 5a and d). IRAK-M knockdown decreased the CXCL10 mRNA expression by cells after JNK inhibitor incubation and TNF-α stimulation (Fig. 5b), while IRAK-M knockdown increased the CXCL10 mRNA expression by cells after p38 MAPK inhibitor incubation and IL-33 stimulation (Fig. 5c).

IRAK-M knockdown had no significant impact on the protein and mRNA expression of CXCL10 in A549 cells incubated with JNK or p38 MAPK inhibitor under IL-33 or HDM stimulation (Additional file 7: Fig. S6c and 6d). IRAK-M knockdown increased the CXCL10 mRNA expression by cells after p38 MAPK inhibitor incubation and IL-1β stimulation (Additional file 7: Fig. S6a), while IRAK-M knockdown increased the CXCL10 mRNA expression by cells after JNK inhibitor incubation and TNF-α stimulation (Additional file 7: Fig. S6b).

### The influence of IRAK-M SNP rs1624395 on serum concentration of CXCL10 in asthma patients

Our previous report showed that asthma susceptible allele G of IRAK-M SNP rs1624395 was associated with a higher expression of IRAK-M mRNA in blood monocytes in asthma patients [7].

**Fig. 6:**
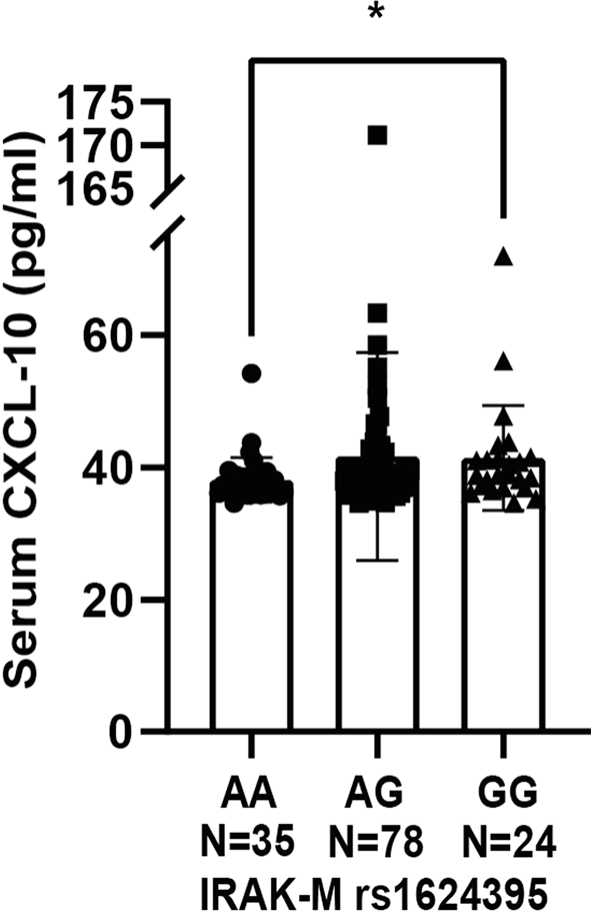
Asthma patients carrying G/G genotypes had higher levels of serum CXCL10 than those carrying homozygote A/A. Serum CXCL10 concentration in asthma patients with different IRAK-M rs1624395 genotypes was detected by ELISA. N = 137 for all asthma patients. The results were assayed in duplicate. Data were presented as mean ± SD, *P < 0.05

In this present study, we found that IRAK-M regulated CXCL10 secretion in lung epithelial cells. Therefore, we tested whether IRAK-M polymorphisms had impact on the serum concentrations of CXCL10 in individual asthmatics. Serum concentration of CXCL10 and genotyping of IRAK-M rs1624395, which is completely interlocked with rs1370128, were performed in 137 individual patients with asthma (Table 1). We demonstrated that asthma patients carrying homozygote G/G had significantly higher levels of serum CXCL10 than those carrying homozygote A/A (41.4 ± 7.8 pg/ml vs 38.1 ± 3.3 pg/ml, p = 0.03) (Fig. 6).

**Table 1:** Clinical and demographic characteristics of the patients

rs1624395/rs1370128	AA/TT	AG/TC	GG/CC
N	35	78	24
Mean age (y)	46.7 ± 16.4	45.1 ± 14.7	41.7 ± 14.7
Male, n (%)	16 (45.7)	24 (30.8)	11 (45.8)
Blood LnEos	5.1 ± 2.1	4.3 ± 2.4	5.5 ± 0.7
Serum LnIgE	4.2 ± 1.9	4.1 ± 2.3	5.5 ± 1.8
Pulmonary function measures			
FEV_1_ (L)	2.1 ± 1.1	2.1 ± 1.1	2.4 ± 1.0
FVC (L)	3.1 ± 1.5	2.8 ± 1.5	3.3 ± 1.3
FEV_1_% pred (%)	65.6 ± 27.3	70.2 ± 36.8	71.7 ± 24.5
FEV_1_/FVC (%)	59.5 ± 22.8	61.4 ± 29.0	66.3 ± 17.9
ICS, n (%)	20 (57.1)	50 (64.1)	13 (54.2)
CXCL10 (ng/ml)	38.1 ± 3.3	41.6 ± 15.6	41.4 ± 7.8

Eos: eosinophil; LnEos: loge of blood eosinophil; LnLgE: loge of total serum IgE; FEV_1_: forced expiratory volume in one second; FVC: forced vital capacity; ICS: inhaled corticosteroids

## Discussion

One of important functions of airway epithelium is to maintain airway homeostasis, which is balanced with potentially deleterious inflammatory effects [2]. Many of identified asthma susceptibility genes, including IRAK-M, are expressed by airway epithelial cells, and dysregulated airway epithelial function is thought to be critical for controlling disease induction [6, 15]. Upon stimulation with aeroallergens, airway epithelial cells, via TLRs (including TLR 2 and 4), release the early epithelial-derived cytokine and chemokines leading to the activation of innate immunity that mediate the type 2 inflammatory responses in the airways [16, 17].

The expression of human IRAK-M of TLR signal pathway is not limited to monocytes, macrophages, but also found in airway epithelial cells and alveolar epithelial cells [6]. Current literature points out the importance of IRAK-M in regulation of epithelial innate immunity [10]. Significant induction of IRAK-M expression was observed in airway epithelial cells during asthmatic process [5, 7, 10, 12, 13]. In this study, using an airway epithelial BEAS-2B cell line and alveolar malignancy-derived A549 cells line, we investigated the effect of IRAK-M with RNA interference on inflammatory response in lung epithelial cells. We exposed the cells to the following typical stimuli: (1) the early response proinflammatory cytokines IL-1β and TNF-α; (2) an epithelial-released cytokine IL-33 that induced airway inflammation via IRAK-M [5]; (3) an environmental aeroallergen HDM triggering allergic inflammatory response via airway-epithelium expressing TLR [16]. We found that individual exposure to IL-1β, or TNF-α, or IL-33, or HDM significantly induced IRAK-M expression at mRNA and protein levels in both BEAS-2B and A549 cells in a time-dependent manner.

Airway epithelial cells play an instructive role in shaping innate or adaptive immune responses during asthmatic process by releasing multiple chemokines and cytokines [1]. By silencing IRAK-M expression in BEAS-2B and A549 cells, we demonstrated IRAK-M knockdown increased the production of proinflammatory cytokines and chemokines, including IL-6 and IL-8, CXCL10 and CXCL11, at both mRNA and protein levels, in response to a variety of stimuli (including IL-1β, TNF-α, IL-33, and HDM). IL-6 and IL-8 are two classic cytokines that are induced by NF-κB with important roles in innate immunity. Systemic elevation of IL-6 was seen in individuals with an exacerbation-prone asthma [18] and increased production of IL-8 by bronchial epithelium cells involved in neutrophilic inflammation in asthmatic process [19].

In this study, to the best of our knowledge, we first reported that IRAK-M regulated expression of chemokine receptor CXCR3 ligands CXCL10 and CXCL11, not CXCL9, by airway epithelial cells upon inflammatory stimulation. CXCL10, the most studied in airway inflammation [20], is a major chemokine produced by bronchial cells in response to several pathogens, such as RV, respiratory syncytial virus and HDM allergen [21–23]. Activated airway epithelial cells are the major source of CXCL10 in the lungs [24], which has been shown to be involved in both bronchial inflammation and airway hyper-responsiveness in a mouse model of asthma [25].

CXCL10 is thought to be induced by Th1 cytokine IFN-γ in a variety of cells that promotes Th1-biased airway inflammation [21]. However, our findings indicated that CXCL10 production by lung epithelial cells was not concomitant with IFN-γ elevation, supporting the previous study showing CXCL10 expression is partially independent of IFN-γ in an OVA-challenged asthma mouse model [25, 26]. A previous study has shown that IRAK-M deficiency leads to excessive activation of p38 MAPK and NF-κB pathways [27], which is consistent with our observation showing overexpression of p-IκBα, p-JNK, and p-p38 MAPK in stimulated IRAK-M KO dendritic cells (DCs) [12]. Moreover, bone marrow monocytes (BMMs) deficient in IRAK-M treated with a toll-like receptor 1/2 (TLR1/2) agonist Pam3Csk increased phosphorylation of p38 MAPK [28]. We found that IRAK-M silencing partly, but significantly, upregulated activation of JNK and p38 MAPK pathways in BEAS-2B and A549 cells after several inflammatory stimuli. Our data was supported by the previous investigation demonstrating p38 MAPK inhibitor SB203580 inhibited the activity but not the activation of p38 MAPK [29]. The effect of IRAK-M on CXCL10 expression was counteracted by JNK inhibitor SP600125 and p38 MAPK inhibitor SB203580 incubation. Our present study suggested alternative pathways that involve the CXCL10 upregulation in lung epithelial cells. Therefore, we inferred that upon stimulation, IRAK-M might involve CXCL10 secretion by lung epithelial cells partly through alternative JNK and p38 MAPK pathways. Our data supported the previous reports showing that significantly less release of CXCL10 from BEAS-2B-eosinophil coculture by blockade of p38 MAPK with its specific inhibitor [30]. These results might indicate a novel pathway that explains the regulatory mechanism of lung epithelial production of CXCL10 in initiation and progression of asthma independent of IFN-γ.

Asthma pathogenesis is caused by gene and environment interaction. To further support this notion, a meta-analysis of pathway enrichment showed that many of the dysregulated genes expressed by nasal or epithelia were linked to asthma pathogenesis [15]. Our previous study performed in a Chinese population had identified two asthma susceptible SNPs, rs1624395 and rs1370128, in IRAK-M locus that were associated with mRNA expression of IRAK-M by circulating monocytes [7]. In this study, we further provided evidence that CXCL10 secretion by lung epithelial cells was partly associated with IRAK-M silencing upon the inflammatory stimulation. Moreover, we found IRAK-M SNPs rs1624395 and rs1370128 both influenced the serum concentrations of CXCL10 in patients with adult asthma. Asthma patients carrying homozygote G/G of rs1624395 had a significantly higher level of serum CXCL10 than those carrying homozygote A/A. CXCR3 chemokines (including CXCL10) secretion has been shown to be regulated by activation of TLRs [31]. IRAK-M negatively regulates TLR signaling [4], thus, it was reasonable that IRAK-M silencing increased CXCL10 expression as presented by our data. These findings implicate that IRAK-M SNPs have an important role in influencing CXCL10 production in asthma patients that should be further investigated in future study.

There are some limitations in our study. First, our study focused only on BEAS-2B and A549 cells as an in vitro model because the scope of our experiments needs cell lines that grow robustly and there is a substantial literature underpinning BEAS-2B and A549 cells use in inflammation research [8, 32, 33]. Because BEAS-2B and A549 cells are both cell lines, our in vitro results might not completely reflect the real status of bronchial epithelial cells in vivo. Differences in IRAK-M expression between BEAS-2B cells and A549 cells could lead to inconsistent results after stimulation between the two cell lines. Samples from bronchial epithelial cells of asthma patients may represent the real effect of IRAK-M on airway inflammatory responses. Secondly, short-term exposure of inflammatory stimuli might not produce chronic inflammatory response in airway epithelial cells. In addition, we only measured the serum concentration of CXCL10 in asthma patients, the most studied and important for asthma pathogenesis among three CXCR3 chemokines [21, 22]. A previous study performed children asthma patients showed the associations between higher serum CXCL10 levels and disease exacerbation and severity [34]. We did observe allele G carriers had a higher level of serum CXCL10, however, we were unable to present the underlying mechanism how IRAK-M SNPs influence the serum level of CXCL10 in this study. Future studies are needed to comprehensively evaluate the expressional changes in the samples, including airway epithelia, sputum, and bronchoalveolar lavage fluid, from asthma patients in the context of genetic architecture.

## Conclusion

We provided evidence that IRAK-M expression was significantly induced in airway epithelial cells after a variety of inflammatory exposure and IRAK-M had multiple effects on inflammatory events of lung epithelial cells with a particular impact on chemokine CXCL10 secretion. Mechanistically, our findings suggest that JNK and p38 MAPK pathways might in part involve the regulation of IRAK-M on CXCL10 secretion by airway epithelial cells. However, we cannot rule out other potential mechanisms of IRAK-M-mediated signaling due to the complexity of IRAK-M signaling pathways. Inhaled corticosteroids (ICSs), the mainstay for asthma therapy, target the established airway inflammation but fail to modulate the underlying mechanisms that drive asthma initiation. Our current observations might suggest a previously unrecognized important role for IRAK-M in regulating cellular inflammation in lung epithelium and indicate that appropriate manipulation of airway-expressing IRAK-M could be a novel target for asthma treatment from disease origin.

## Supplementary Information

**Additional file 1: Table S1**. qRT-PCR primers used in this study.

**Additional file 2: Figure S1.** IRAK-M expression is inducible by multiple stimuli in A549 cells. IRAK-M protein expression at 0, 6, 12, 24 h after (**a**) IL-1β (1 ng/ml), (**b**) TNF-α (10 ng/ml), (**c**) IL-33 (100 ng/ml) and (**d**) HDM (10 μg/ml) exposure. IRAK-M protein expression was normalized to GAPDH. Values are expressed as mean ± SEM (n = 3). *P < 0.05.

**Additional file 3: Figure S2.** IRAK-M expression was attenuated by IRAK-M siRNA in BEAS-2B and A549 cells. (**a**) Expression of IRAK-M at both mRNA level and protein level in BEAS-2B cells after IRAK-M knockdown by siRNAs. (**b**) Expression of IRAK-M at both mRNA level and protein level in A549 cells after IRAK-M knockdown by siRNAs. Both mRNA and protein expression of IRAK-M were normalized to GAPDH. Values are expressed as mean ± SEM (n = 3). *P < 0.05.

**Additional file 4: Figure S3.** Impact of IRAK-M knockdown on cytokines production in A549 cells. Expression of IL-6, IL-8, CXCL10, CXCL11 and IFN-γ at both mRNA level and protein level after (**a**) IL-1β (1 ng/ml), (**b**) TNF-α (10 ng/ml), (**c**) IL-33 (100 ng/ml) and (**d**) HDM (10 μg/ml) stimulation for 24 h. mRNA expression of IL-6, IL-8, CXCL10, CXCL11 and IFN-γ were normalized to GAPDH. Values are expressed as mean ± SEM (n = 3). *P < 0.05. CON, si-control. KD, si-IRAK-M.

**Additional file 5: Figure S4.** Effect of IRAK-M on activation of JNK and p38 MAPK pathways in A549 cells. Protein expression of p-JNK, JNK, p-p38, p38 after (**a**) IL-1β (1 ng/ml), (**b**) TNF-α (10 ng/ml), (**c**) IL-33 (100 ng/ml) and (**d**) HDM (10 μg/ml) stimulation for 10, 30 and 60 min. P-JNK was normalized to total JNK, while p-p38 was normalized to total p38. Values are expressed as mean ± SEM (n = 3). *P < 0.05.

**Additional file 6: Figure S5.** The activation of JNK and p38 MAPK pathway was blocked by inhibitor in A549 cells. (**a**) Protein expression of p-JNK and JNK after SP600125 (SP, 20 μM) pretreated for 2 h and IL-1β stimulated for 24 h. (**b**)Protein expression of p-p38 and p38 after SB203580 (SB, 10 μM) pretreated for 2 h and IL-1β stimulated for 24 h. P-JNK was normalized to total JNK, while p-p38 was normalized to total p38. Values are expressed as mean ± SEM (n = 3). *P < 0.05.

**Additional file 7: Figure S6.** Treatment with JNK or p38 MAPK inhibitor attenuated IRAK-M knockdown-mediated CXCL10 secretion upon stimulation in A549 cells. Expression of CXCL10 at both mRNA and protein level after SP600125 (SP, 20 μM) and SB203580 (SB, 10 μM) incubation for 2 h and (**a**) IL-1β (1 ng/ml), (**b**) TNF-α (10 ng/ml), (**c**) IL-33 (100 ng/ml) and (**d**) HDM (10 μg/ml) stimulation for 24 h. Values are expressed as mean ± SEM (n = 3). *P < 0.05. CON, si-control. KD, si-IRAK-M.

## Data Availability

The datasets supporting the conclusions of this article are included within the article and its additional files. Any other data and materials generated in this study are available from the corresponding author upon request.

## Abbreviations

IL-1β: Interleukin-1beta
TNF-α: Tumor necrosis factor-alpha
CXCL: Chemokine (C-X-C motif) ligand
ELISA: Enzyme-linked immunosorbent assay
GAPDH: Glyceraldehyde 3-phosphate dehydrogenase
mRNA: Messenger ribonucleic acid
OVA: Ovalbumin
PBS: Phosphate-buffered saline
MAPK: Mitogen-activated protein kinase
