# Supplementary material for: IRAK-M has effects in regulation of lung epithelial inflammation
Source: Respir Res. 2023 Apr 7;24:103. doi: 10.1186/s12931-023-02406-5 (PMC10082527; doi:10.1186/s12931-023-02406-5)
Supplement: Supplementary file 1 — Additional file 1: Table S1. qRT-PCR primers used in this study. [file 12931_2023_2406_MOESM1_ESM.docx]

**Table S1** qRT-PCR primers used in this study

| Gene Name | Forward Primer | Reverse Primer |
| --- | --- | --- |
| IRAK-M | 5’-CTGCGGGATCTCCTTAGAGAA-3’ | 5’-GCAGAGAAATTCCGAGGGCA-3’ |
| IL-6 | 5’AAATTCGGTACATCCTCGACGGCA-3’ | 5’AGTGCCTCTTTGCTGCTTTCACAC-3’ |
| IL-8 | 5’-TCCTGATTTCTGCAGCTCTG-3’ | 5’-GTCCACTCTCAATCACTCTCAG-3’ |
| CXCL10 | 5’-GTGGCATTCAAGGAGTACCTC-3’ | 5’-TGATGGCCTTCGATTCTGGATT-3’ |
| CXCL11 | 5’-GACGCTGTCTTTGCATAGGC-3’ | 5’-GGATTTAGGCATCGTTGTCCTTT-3’ |
| IFN-γ | 5’-TCGGTAACTGACTTGAATGTCCA-3’ | 5’-TCGCTTCCCTGTTTTAGCTGC-3’ |
| GAPDH | 5’-GGAGCGAGATCCCTCCAAAAT-3’ | 5’-GGCTGTTGTCATACTTCTCATGG-3’ |

Table II. Demographics of asthma patients

| rs1624395/rs1370128 | AA/TT | AG/TC | GG/CC |
| --- | --- | --- | --- |
| N | 35 | 78 | 24 |
| Mean age (y) | 46.7±16.4 | 45.1±14.7 | 41.7±14.7 |
| Male, n (%) | 16 (45.7) | 24 (30.8) | 11 (45.8) |
| Blood LnEos | 5.1±2.1 | 4.3±2.4 | 5.5±0.7 |
| Serum LnIgE | 4.2±1.9 | 4.1±2.3 | 5.5±1.8 |
| Pulmonary function measures |  |  |  |
| FEV_1_ (L) | 2.1±1.1 | 2.1±1.1 | 2.4±1.0 |
| FVC (L) | 3.1±1.5 | 2.8±1.5 | 3.3±1.3 |
| FEV_1_% pred (%) | 65.6±27.3 | 70.2±36.8 | 71.7±24.5 |
| FEV_1_/FVC (%) | 59.5±22.8 | 61.4±29.0 | 66.3±17.9 |
| CXCL10 (ng/ml) | 38.1±3.3 | 41.6±15.6 | 41.4±7.8 |

Eos, eosinophil; LnEos, loge of blood eosinophil; LnLgE, loge of total serum IgE; FEV_1_, forced expiratory volume in one second; FVC, forced vital capacity.
