# Supplementary figures and images for: IRAK-M has effects in regulation of lung epithelial inflammation
Source: Respir Res. 2023 Apr 7;24:103. doi: 10.1186/s12931-023-02406-5 (PMC10082527; doi:10.1186/s12931-023-02406-5)

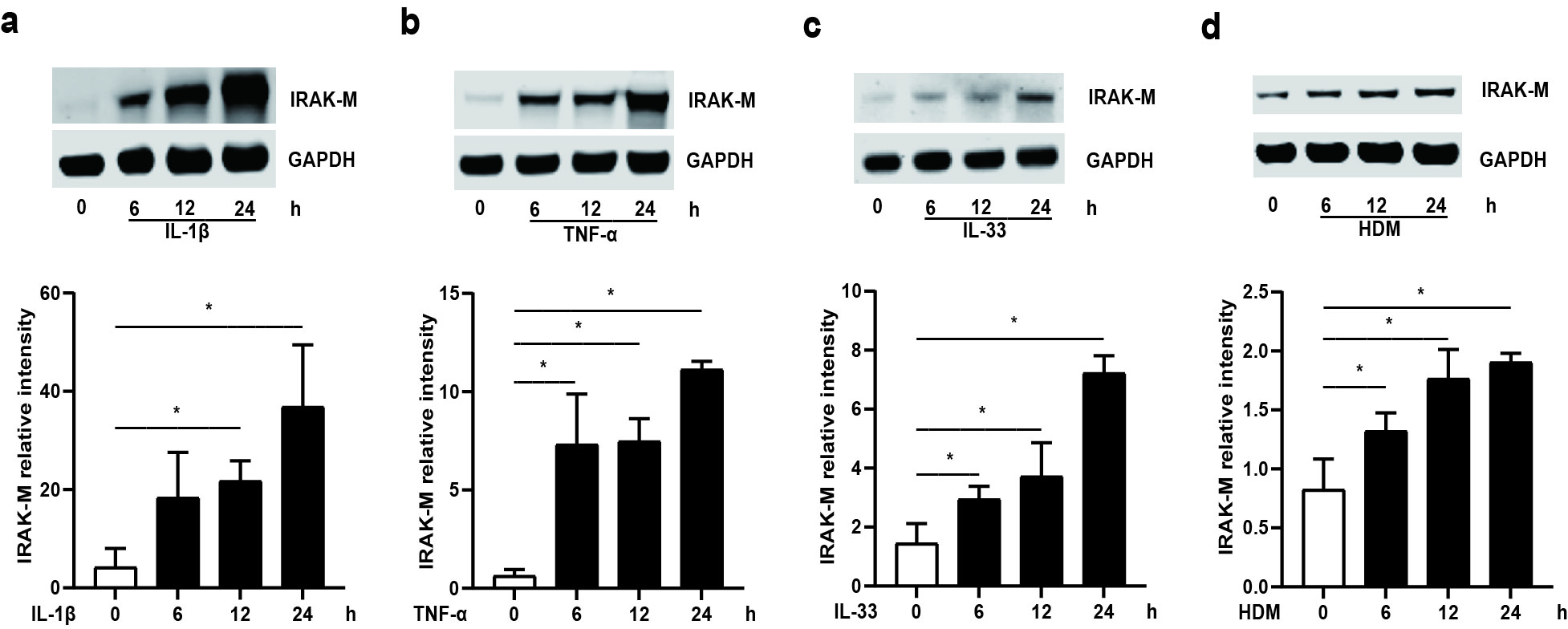

Supplement: Supplementary file 2 — Additional file 2: Figure S1. IRAK-M expression is inducible by multiple stimuli in A549 cells. IRAK-M protein expression at 0, 6, 12, 24 h after (a) IL-1β (1 ng/ml), (b) TNF-α (10 ng/ml), (c) IL-33 (100 ng/ml) and (d) HDM (10 μg/ml) exposure. IRAK-M protein expression was normalized to GAPDH. Values are expressed as mean ± SEM (n = 3). *P < 0.05. [file 12931_2023_2406_MOESM2_ESM.jpg]

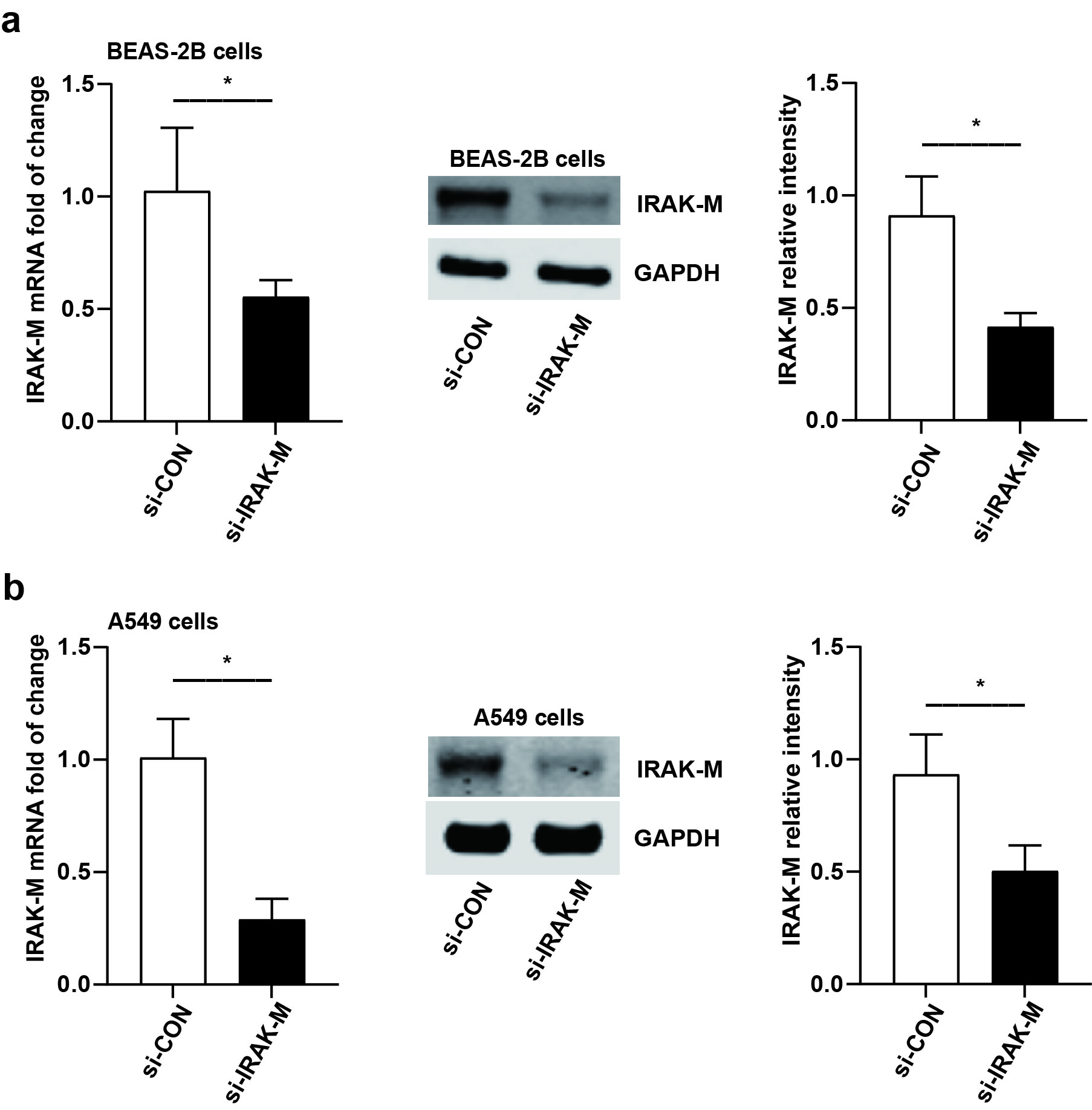

Supplement: Supplementary file 3 — Additional file 3: Figure S2. IRAK-M expression was attenuated by IRAK-M siRNA in BEAS-2B and A549 cells. (a) Expression of IRAK-M at both mRNA level and protein level in BEAS-2B cells after IRAK-M knockdown by siRNAs. (b) Expression of IRAK-M at both mRNA level and protein level in A549 cells after IRAK-M knockdown by siRNAs. Both mRNA and protein expression of IRAK-M were normalized to GAPDH. Values are expressed as mean ± SEM (n = 3). *P < 0.05. [file 12931_2023_2406_MOESM3_ESM.jpg]

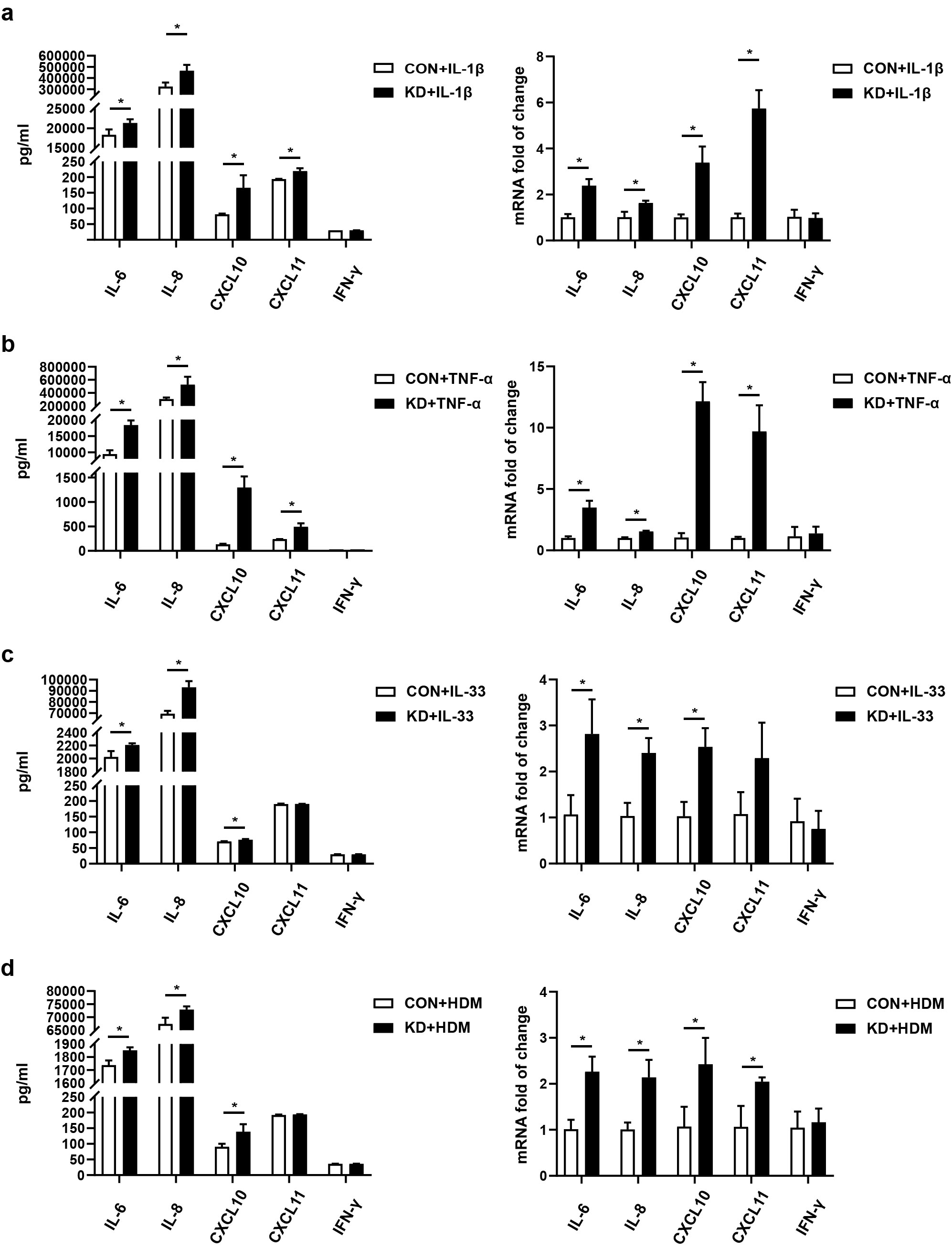

Supplement: Supplementary file 4 — Additional file 4: Figure S3. Impact of IRAK-M knockdown on cytokines production in A549 cells. Expression of IL-6, IL-8, CXCL10, CXCL11 and IFN-γ at both mRNA level and protein level after (a) IL-1β (1 ng/ml), (b) TNF-α (10 ng/ml), (c) IL-33 (100 ng/ml) and (d) HDM (10 μg/ml) stimulation for 24 h. mRNA expression of IL-6, IL-8, CXCL10, CXCL11 and IFN-γ were normalized to GAPDH. Values are expressed as mean ± SEM (n = 3). *P < 0.05. CON, si-control. KD, si-IRAK-M. [file 12931_2023_2406_MOESM4_ESM.jpg]

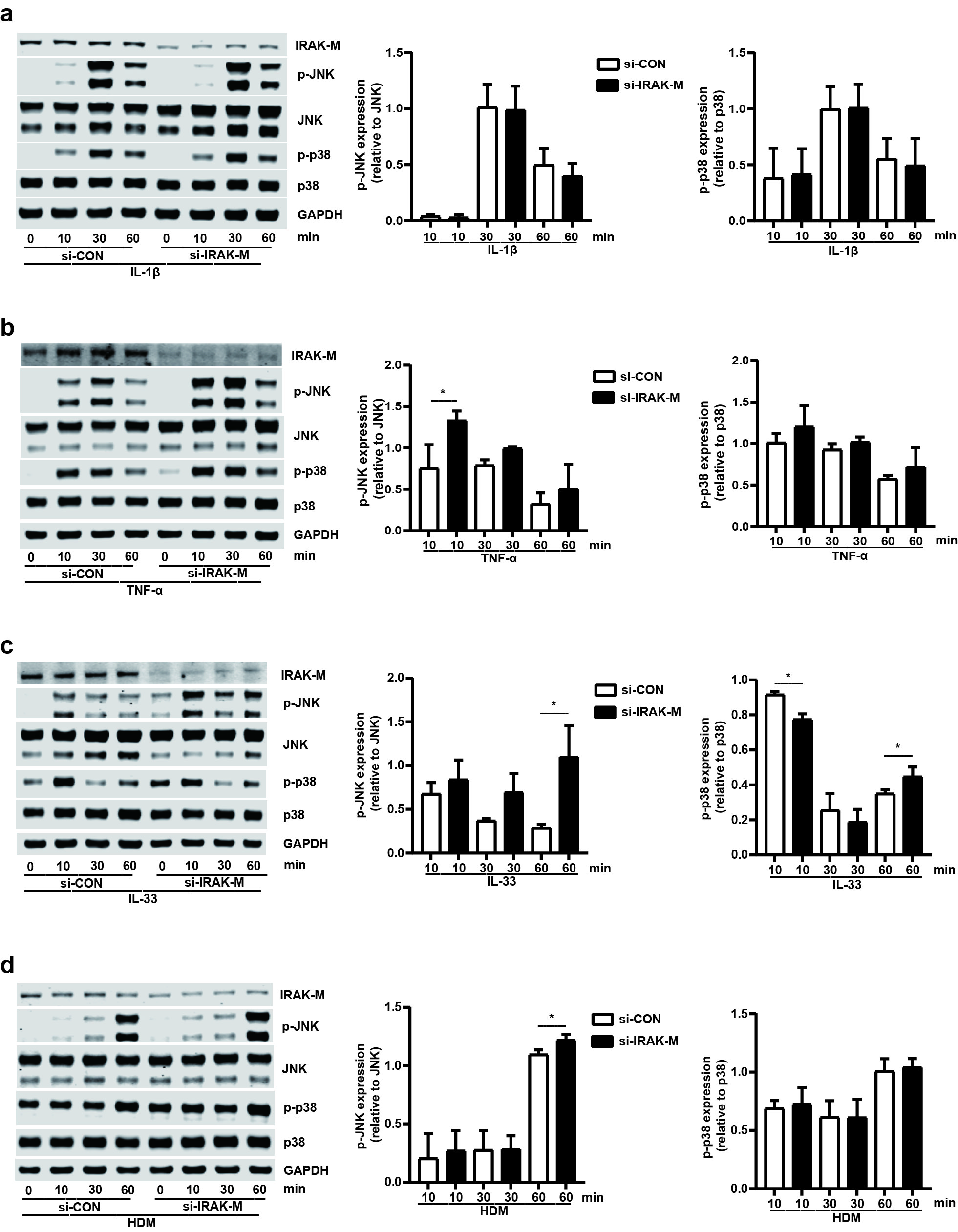

Supplement: Supplementary file 5 — Additional file 5: Figure S4. Effect of IRAK-M on activation of JNK and p38 MAPK pathways in A549 cells. Protein expression of p-JNK, JNK, p-p38, p38 after (a) IL-1β (1 ng/ml), (b) TNF-α (10 ng/ml), (c) IL-33 (100 ng/ml) and (d) HDM (10 μg/ml) stimulation for 10, 30 and 60 min. P-JNK was normalized to total JNK, while p-p38 was normalized to total p38. Values are expressed as mean ± SEM (n = 3). *P < 0.05. [file 12931_2023_2406_MOESM5_ESM.jpg]

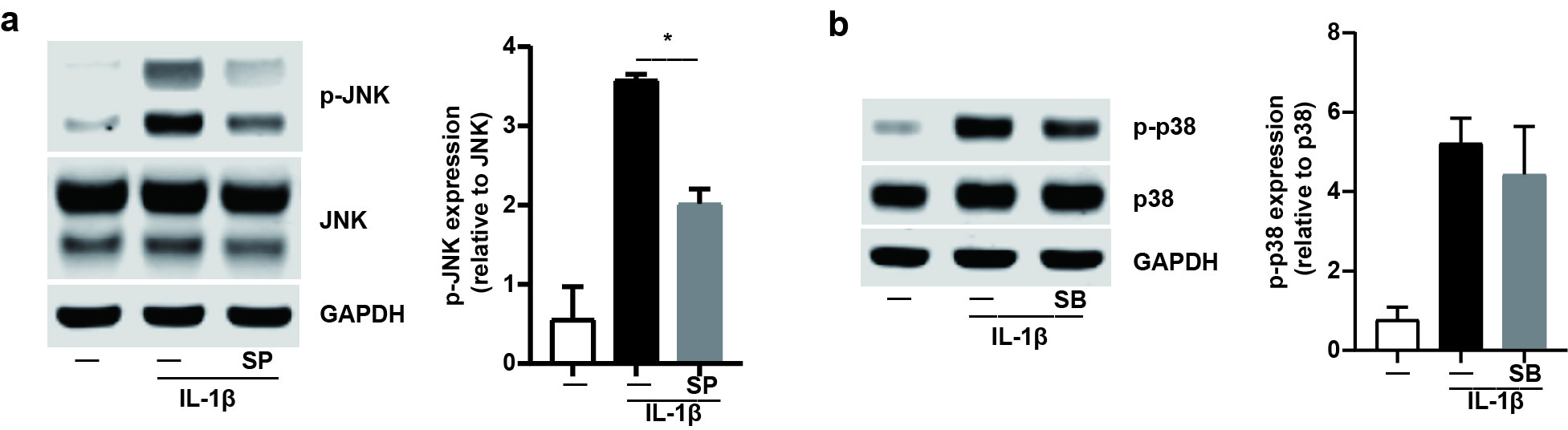

Supplement: Supplementary file 6 — Additional file 6: Figure S5. The activation of JNK and p38 MAPK pathway was blocked by inhibitor in A549 cells. (a) Protein expression of p-JNK and JNK after SP600125 (SP, 20 μM) pretreated for 2 h and IL-1β stimulated for 24 h. (b)Protein expression of p-p38 and p38 after SB203580 (SB, 10 μM) pretreated for 2 h and IL-1β stimulated for 24 h. P-JNK was normalized to total JNK, while p-p38 was normalized to total p38. Values are expressed as mean ± SEM (n = 3). *P < 0.05. [file 12931_2023_2406_MOESM6_ESM.jpg]

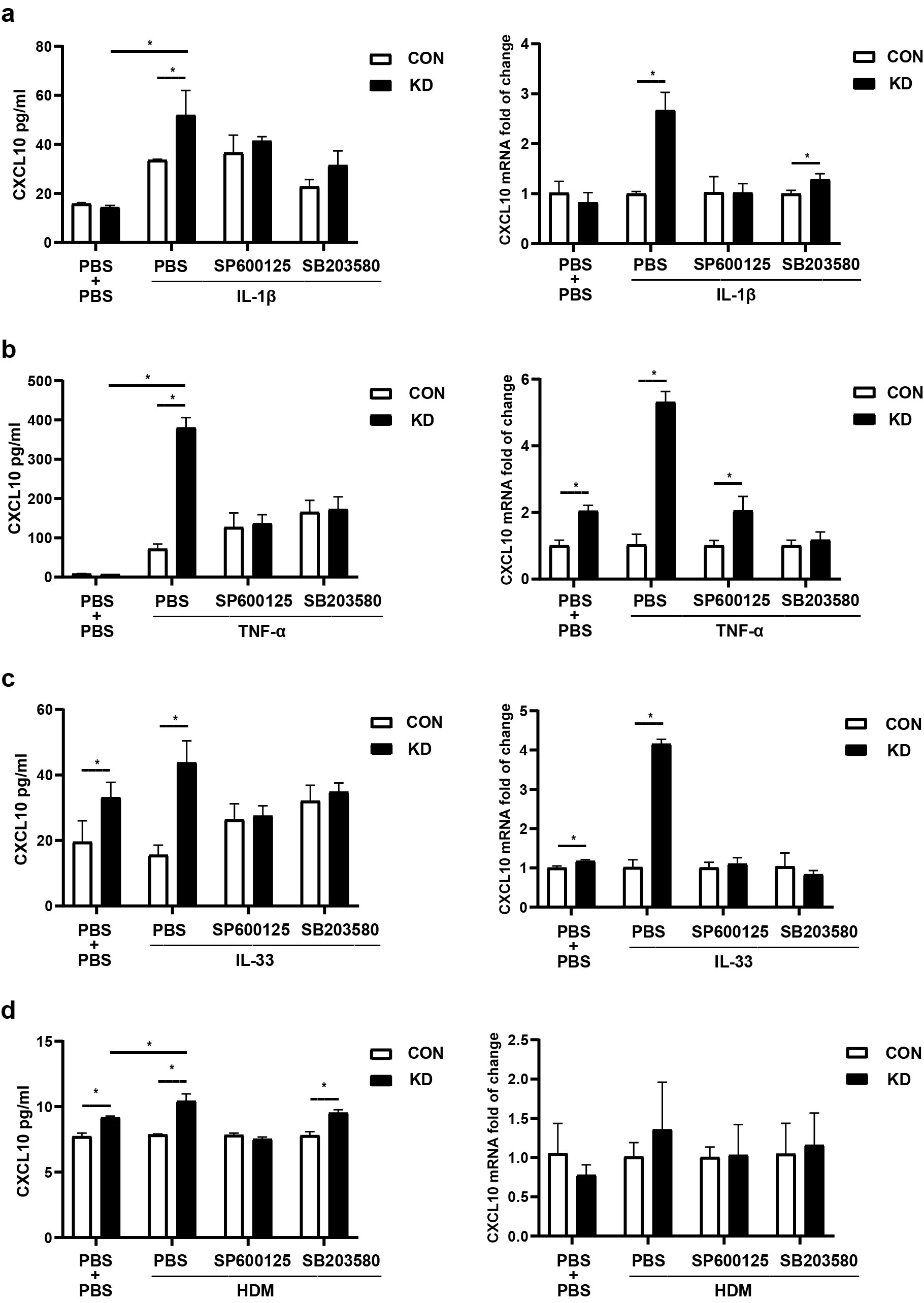

Supplement: Supplementary file 7 — Additional file 7: Figure S6. Treatment with JNK or p38 MAPK inhibitor attenuated IRAK-M knockdown-mediated CXCL10 secretion upon stimulation in A549 cells. Expression of CXCL10 at both mRNA and protein level after SP600125 (SP, 20 μM) and SB203580 (SB, 10 μM) incubation for 2 h and (a) IL-1β (1 ng/ml), (b) TNF-α (10 ng/ml), (c) IL-33 (100 ng/ml) and (d) HDM (10 μg/ml) stimulation for 24 h. Values are expressed as mean ± SEM (n = 3). *P < 0.05. CON, si-control. KD, si-IRAK-M. [file 12931_2023_2406_MOESM7_ESM.jpg]
